# Correspondence

**Published:** 1877-03

**Authors:** Otto Arnold


### CORRESPONDENCE.

IRONTON, OHIO, February 17, 1877.

DR. J. TAFT—DEAR SIR:—I herewith submit you the following case occurring in my practice: Mrs. B——, aged forty, called to consult me in reference to a "tumor" of seven months growth upon her gum. Examining her mouth, I

discovered a "tumor" about the size of an egg upon the left lower alveolus, extending from the first bicuspid tooth back to the ramus, and laterally to such an entent as to push her tongue to one side, materially interfering with speech and mastication; back of the bicuspid, on the affected side, the teeth were all decayed away, the roots only remaining; to the touch the growth was quite firm and elastic, considering its rapid development. Texture and general appearance of patient: her complexion being decidedly sallow, the growth was pronounced a scirrhus tumor, and an operation advised for its removal. Five days later the patient was etherized; the soft parts or tumor excised; remaining roots extracted; carious portion of the bone removed with bone forceps and scrapers, and sulphuric acid, applied full strength, to dissolve away any remaining portion of carious bone. After the operation patient was able to walk to her boarding house without assistance. The next day after operation patient was, "with the exception of a slight weakness," feeling quite comfortable, having been able to rest well the night following the operation without an opiate. Prescribed iodine and iodide of potassa internally, and a mouth wash of salicylic acid, and after another application of sulphuric acid 'dilut.' on the second day after operation, she was allowed to go home, she living in the country. Ten days after operation, patient presented herself expressing great satisfaction that the operation was a success, as every appearance indicated the parts operated upon were in a healthy healing condition, while the time has been too short to arrive at any satisfactory conclusions. I have no doubt, if patient continues iodine treatment, that the tumor will not again recur. Very respectfully yours, etc., OTTO ARNOLD.

